# Assessment of the relationship between labial gingival thickness and the underlying bone thickness in maxillary anterior teeth by two digital techniques

**DOI:** 10.1038/s41598-021-04721-7

**Published:** 2022-01-13

**Authors:** Linhong Wang, Yan Ruan, Jianping Chen, Yunxiao Luo, Fan Yang

**Affiliations:** 1 Health Management Center, Department of Stomatology, Zhejiang Provincial People’s Hospital, Affiliated People’s Hospital, Hangzhou Medical College, Hangzhou, 310014 Zhejiang People’s Republic of China; 2 Department of Stomatology, Bengbu Medical College, Bengbu, 233030 Anhui People’s Republic of China; 3 Department of Stomatology, Zhejiang Chinese Medical University, Hangzhou, 310053 People’s Republic of China

**Keywords:** Biological techniques, Anatomy

## Abstract

This study aimed to noninvasively assess the relationship between the labial gingival thickness (GT) and the underlying bone thickness (BT) of maxillary anterior teeth by two digital techniques. A total of 30 periodontally healthy participants with 172 maxillary anterior teeth were enrolled. GT and BT were measured at 2, 4 and 6 mm apical to the cemento-enamel junction (CEJ) by two digital techniques: M1—cone-beam computed tomography (CBCT) and M2—digital intraoral scanning (DIS) combined with CBCT. The Pearson's correlation coefficient was calculated to determine the correlation between GT and BT. A significant negative correlation was identified between GT and BT at 2 mm apical to the CEJ for central incisors (CI), lateral incisors (LI), and canines (CA) both by M1 and M2, while a weak negative correlation at 4 mm apical to the CEJ was observed by M1 for CA. No significant correlation was found at other sites by both M1 and M2. The labial BT was < 1 mm in most cases (85% of CI; 97% of LI; and 90% of CA). Within the limitation of this study, it was concluded that GT and BT seemed to be negatively correlated at 2 mm apical to the CEJ. Therefore, caution is warranted when implant restoration at the esthetic area of the anterior teeth.

## Introduction

The gingival biotype (GB) is closely associated with the long and short-term esthetic outcomes of implant placement, restorative treatments, and periodontal therapy in the esthetic region of the anterior maxilla. Especially in cases of immediate implantation, the implant must be placed in the ideal three-dimensional position with a thick GB and a thick labial bone plate^1^. A complete labial bone plate with a thickness of at least 1–2 mm is vital for preventing bone resorption on the labial side of the implant^2,3^, as well as to ensure the implant's stability and long-term esthetic outcome of the soft tissue. Moreover, the gingival thickness is another factor affecting the long-term outcome of anterior esthetic effects^4,5^. Compared with thick GB, patients with thin GB are more likely to develop gingival recession and alveolar bone retraction after implantation, which is associated with a high risk of esthetics^6^. Therefore, gingival thickness (GT) and the underlying alveolar bone thickness (BT) in the esthetic zone seem to play a decisive role in treatment outcomes.

Accurate measurement of GT and the underlying BT before implant placement is pivotal to prevent complications such as soft tissue recession and implant exposure^7,8^. However, limited information is available regarding the relationship between the labial BT and GT due to the lack of a standardized measurement technique for simultaneously measuring the thickness of hard and soft tissues^9^. Regrettably, there are relatively few studies on this issue. Whether GT is specifically correlated with the thickness of the labial bone is still controversial, as no consensus has been reached so far^10,11^. There are numerous methods to measure GB, GT, and BT, such as transgingival probing with periodontal probe^10,12^, which observe GB by the outline of the periodontal probe through the soft tissue. However, reports on the accuracy of this method vary in different studies^10–12^. Methods for measuring GT include invasive ones such as direct puncture, which requires local anesthesia^13^, and non-invasive methods such as ultrasonic measurement. However, their accuracies are limited for determining GT in some areas such as the posterior teeth^14^. The cone-beam computed tomography (CBCT) is an objective approach for determining the thickness of soft and hard tissues^15^, and some reports have indicated that CBCT has high precision in measuring BT^16,17^. Nonetheless, its low resolution and contrast limit the visualization of soft tissues^11,18–20^.

The limitations of the above methods for measuring GT and BT warrant further studies in this area. Digital intraoral scanners (DIS) enable the acquisition of data directly from the oral cavity and have been widely used in dentistry^21^. DIS images with more accurate outlines and greater resolution than CBCT were applied to measure the volume of periodontal tissues^22^ and exhibited higher precision and reliability^23^. DIS enhances reproducibility and reduces the variance in measurements between different investigators. Several studies have established the accuracy of DIS on single elements or part of the arch commonly used in restorative or prosthodontic dentistry, such as crowns, inlays, onlays, and short bridges^24–28^. In contrast, the accuracy of DIS in multiple units or the full arch is still questionable^29^. The precision was clinically acceptable when scanning less than half of the arch^30^. However, no consensus was reached on the accuracy of DIS for soft tissues so far^31,32^. DIS data can be combined with CBCT data. Whether the measurement of GT and BT on the superimposed data generated by CBCT and DIS will be more precise remains to be elucidated.

It is pivotal to clarify the correlation between GT and BT. The purpose of this study was to analyze the correlation between the thickness of the gingiva and the underlying bone in the esthetic area of maxillary anterior teeth through two digital techniques: CBCT (M1) and DIS combined with CBCT data (M2) to provide a theoretical basis for the clinical selection of esthetic restoration and implant treatment in clinical practice.

## Materials and methods

### Sample selection

Thirty medical students (18 males and 12 females) aged 20 to 26 years in Zhejiang Provincial People's Hospital were enrolled in this study. Informed consent forms were signed by the participants, and this study was approved by the Ethics Committee of Zhejiang Provincial People's Hospital (2018KY015) and performed according to the principles of the Declaration of Helsinki.

Inclusion criteria: all subjects had a healthy periodontal condition with a periodontal probing depth of no more than 3 mm, a bleeding index of ≤ 2 (according to the bleeding index standard proposed by Mazza in 1981), and no gingival recession in maxillary anterior teeth.

Exclusion criteria: pregnancy or lactating females; fillings or crowns in the maxillary anterior dentition; tooth malocclusion; use of any medication affecting soft tissues; cigarette smoking; and history of orthodontic therapy. Each subject was given instructions on maintaining oral hygiene and had their teeth cleaned one week before the test. As a result, 172 teeth were included in the study (4 CIs and 4 LIs were excluded due to caries, periapical diseases, or torsional teeth).

### Method 1 (M1): CBCT measurements

**Figure 1:**
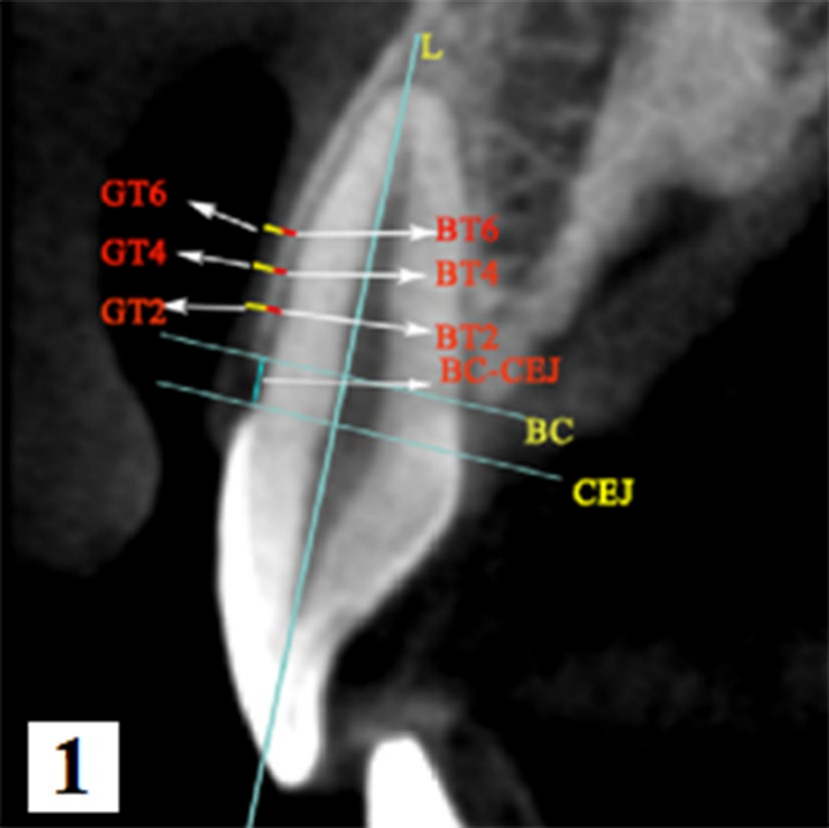
Measurements were taken in the median sagittal plane of teeth with a simple CBCT: distance from the bone crest to the CEJ (BC-CEJ); gingival thickness (GT2, GT4, and GT6) at 2, 4, and 6 mm apical to the CEJ; labial bone thickness (BT2, BT4, and BT6) at 2, 4, and 6 mm apical to the CEJ.

GT and BT were assessed using CBCT (ProMax 3D, Planmeca, Helsinki, Finland) by an experienced dental radiologist. Before scanning, the lips and cheeks were retracted by a sterile plastic retractor. All scans were performed at 80 kV and 6.0 mA for 15 s (voxel size: 0.15 mm; grayscale: 15 bits; focal spot: 0.5 mm; and field of view: 12 × 9 cm). Image reconstruction for visual analysis was performed by the Romexis software (Romexis Viewer 3.5.1R, Planmeca, Helsinki, Finland). Measurements of the labial BT and GT for each tooth were performed at 2, 4, and 6 mm apical to the CEJ on the mid-labial aspect perpendicular to the axis of the tooth in the sagittal plane (Fig. 1). All sites were measured by the same clinician. To assess intra-examiner reliability, duplicate registration was performed by the same examiners at an interval of 24 h.

### Method 2 (M2): DIS + CBCT measurements

**Figure 2:**
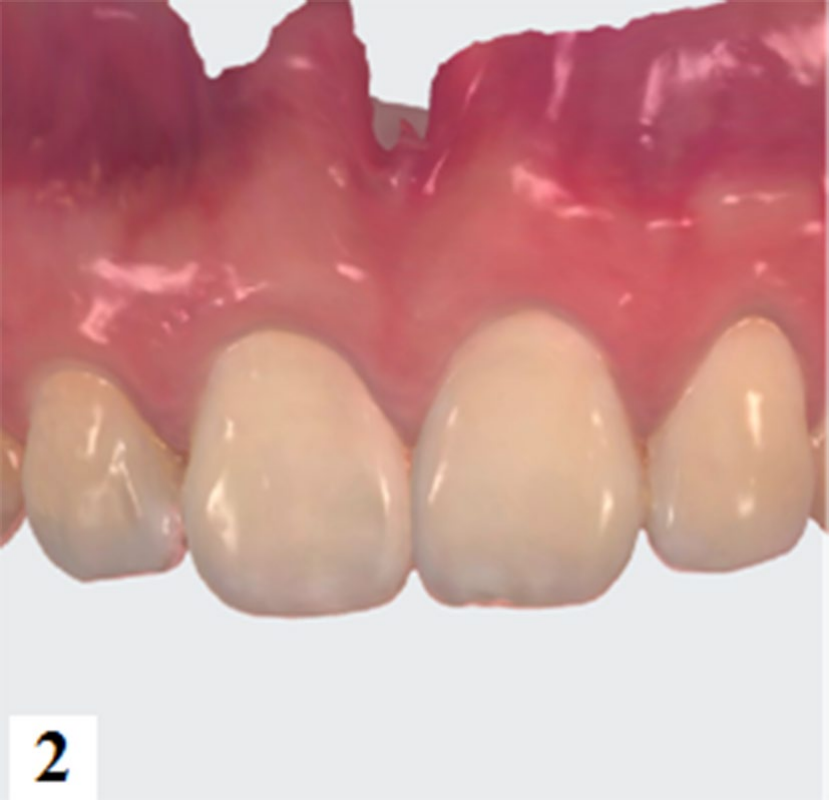
Intraoral soft tissue images were collected by TRIOS intraoral scanner.

**Figure 3:**
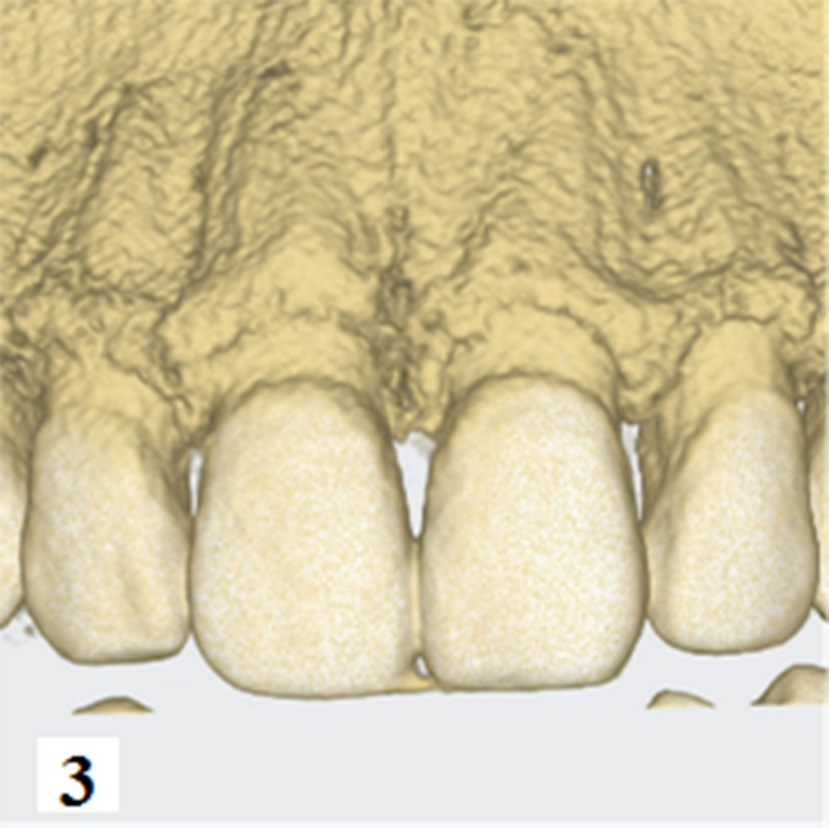
Hard tissue images were taken by CBCT.

**Figure 4:**
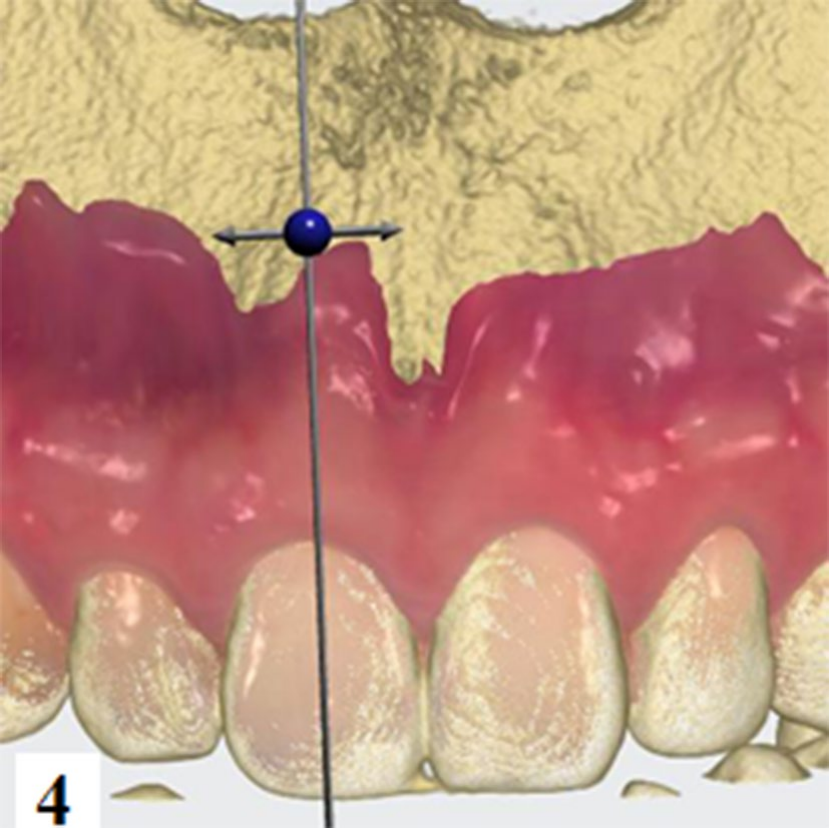
The DIS data were exported into stereolithography (STL) format and matched with the CBCT data, measurements were taken along the median sagittal plane of the tooth.

**Figure 5:**
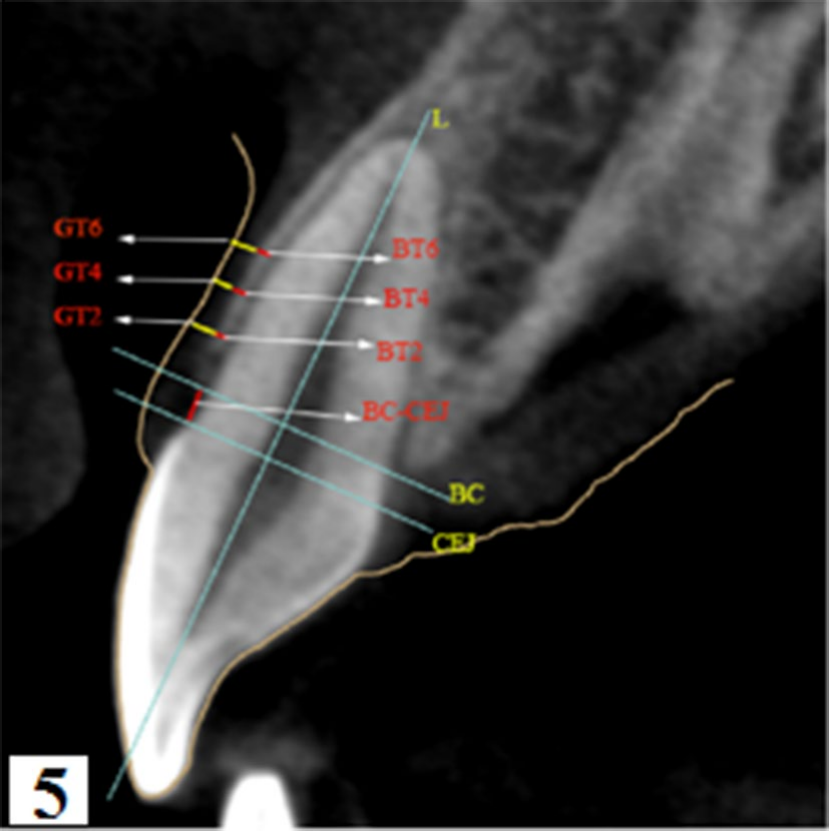
The scanned gingival profile was automatically marked with a thin yellow line. Measurement of the distance from the bone crest to the CEJ (BC-CEJ); gingival thickness (GT2, GT4, and GT6) at 2, 4, and 6 mm apical to the CEJ; labial bone thickness (BT2, BT4, and BT6) at 2, 4, and 6 mm apical to the CEJ.

GT and BT were assessed after registration between oral soft tissue images obtained by DIS and hard tissue images generated by CBCT. The teeth and gingival surfaces were cleaned and dried before intraoral scanning^22^. The complete maxillary dentition, labial and palatal gingival tissues were scanned by an experienced technician using an intraoral scanner TRIOS (3Shape, Copenhagen, Denmark). The DIS data were exported into stereolithography (STL) format and matched with the CBCT data. Three highly radiopaque and relatively stable identical anatomical sites on the teeth were chosen as fiducial markers and were used as references to match the STL files with the CBCT images (acquired in M1) according to a best-fit algorithm using the TRIOS software (Implant Studio, 3Shape, Copenhagen, Denmark). The soft tissue outline on the labial side was visible as a yellow line. GT was defined as the distance from the surface of the gingiva to the surface of the alveolar bone or the surface of the tooth (Figs. 2, 3, 4, 5).

Labial GT and BT measurements were performed at 2 mm, 4 mm, and 6 mm apical to the CEJ at the mid-buccal aspect of each single-rooted tooth and perpendicular to the axis of the tooth (L). Furthermore, the distance from the CEJ to the alveolar bone crest (BC) was also measured (BC-CEJ) (Figs. 1 and 5). All the clinical procedures and measurements were performed by the same clinician. To assess intra-examiner reliability, duplicate registration was performed by the same examiners at an interval of 24 h.

### Statistical analysis

The data was analyzed by SPSS 26.0 (Statistical Product and Service Solutions 26.0 Inc, Chicago, IL, USA). The normality of data distribution was evaluated by the Shapiro–Wilk test. Data subject to a normal distribution were expressed as mean ± standard deviation (${\overline{\text{x}}} \pm {\text{s}}$). The intra-group correlation coefficient (ICC) was used to evaluate the consistency of examiners. Differences between M1 and M2 were analyzed by the paired t-test. The difference between BT and GT among CI, LI, and CA were compared by analysis of variance of randomized block design. SNK was used for post pairwise comparisons. Pearson correlation analysis was used to analyze the correlation between GT and BT among each tooth type. The judgment criteria of relevant strengths were as follows: ① Mild correlation: 0.00 < *r* < 0.39; ② Moderate correlation: 0.40 < *r* < 0.69; ③ Height correlation: 0.70 < *r* < 1.00. Significance level: α = 0.05.

## Results

**Figure 6:**
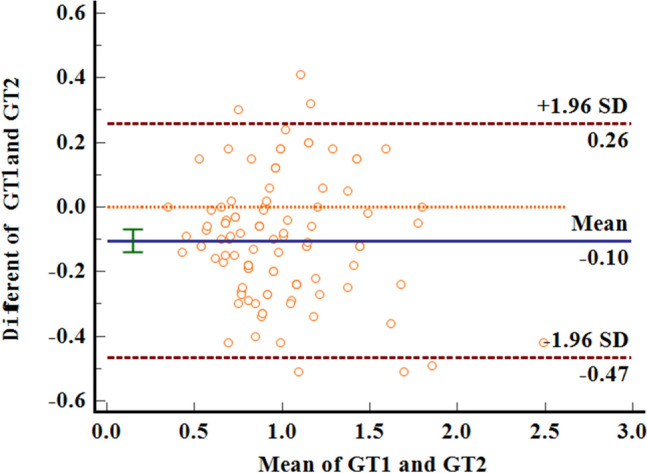
The Bland–Altman plots of GT measured by the same examiner at an interval of 24 h.

**Figure 7:**
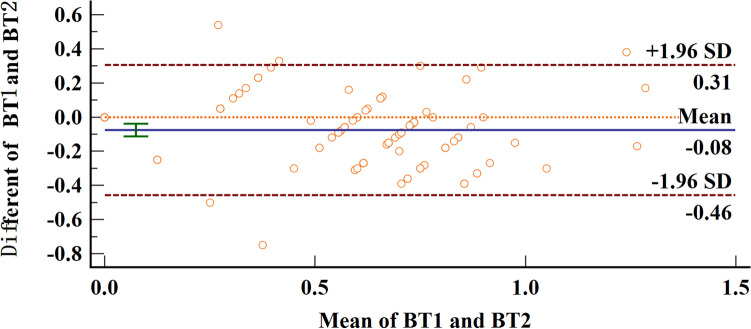
The Bland–Altman plots of BT measured by the same examiner at an interval of 24 h.

172 anterior teeth of 30 participants with a mean age of 24 years were included. Two sets of recordings for M1 and M2 measurements of GT and BT were performed by the same examiner at an interval of 24 h to evaluate intra-examiner reliability. The ICC values of duplicate recordings for GT was 0.813 (95% CI 0.738, 0.866) and 0.865 (95% CI 0.809, 0.906) for BT (Table 1). The result of Bland–Altman plots are showed in Figs. 6 and 7. Duplicate records demonstrated optimal consistencies between the two times.

**Figure 8:**
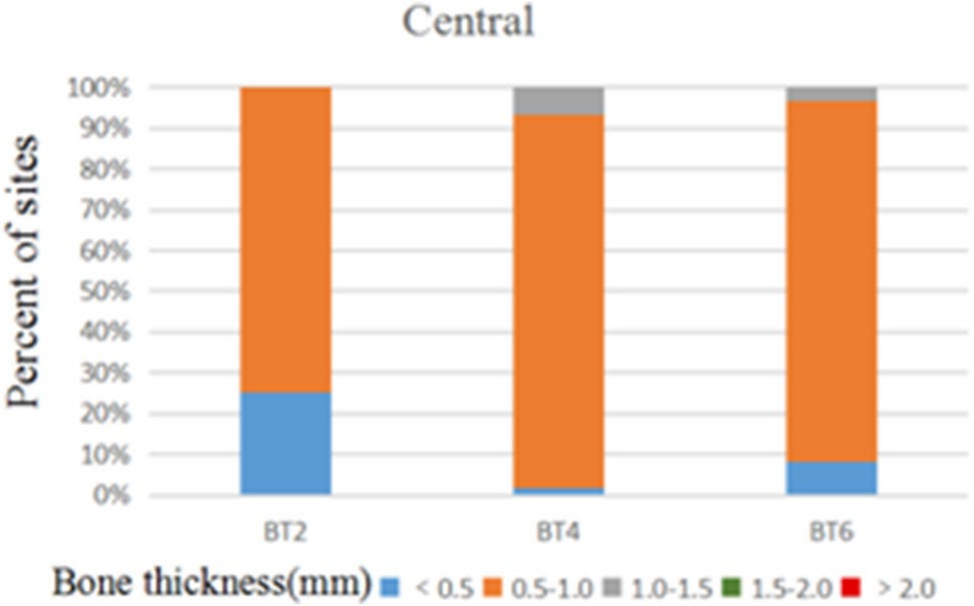
Distribution frequency of bone thickness at different sites in the central incisor region.

**Figure 9:**
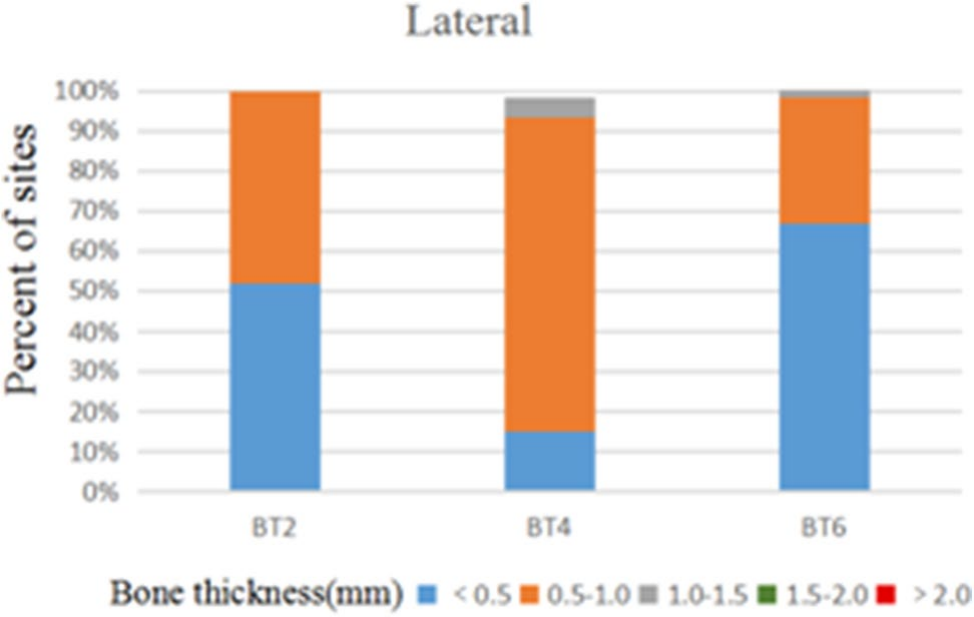
Distribution frequency of bone thickness at different sites in the lateral incisor region.

**Figure 10:**
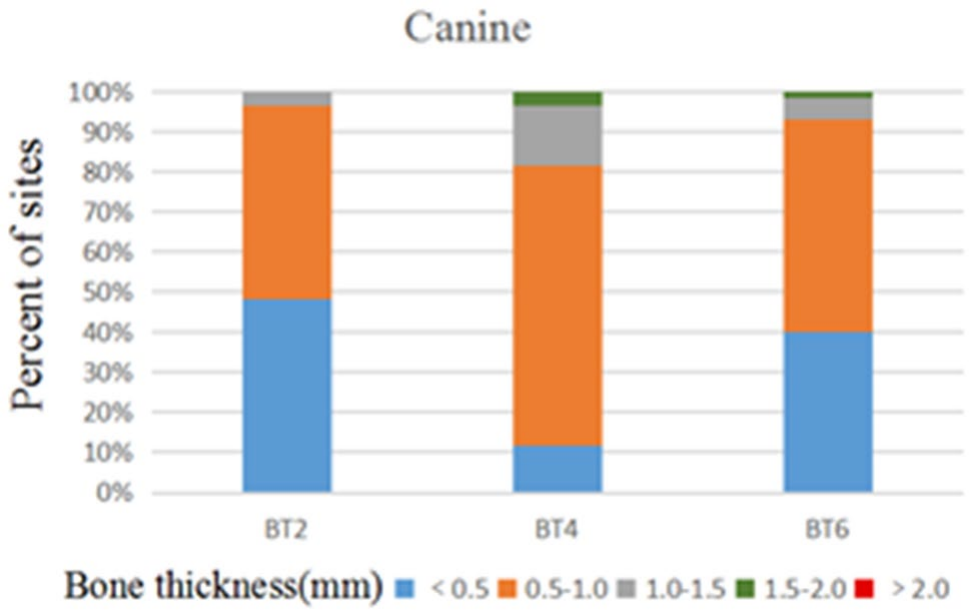
Distribution frequency of bone thickness at different sites in the canine region.

**Figure 11:**
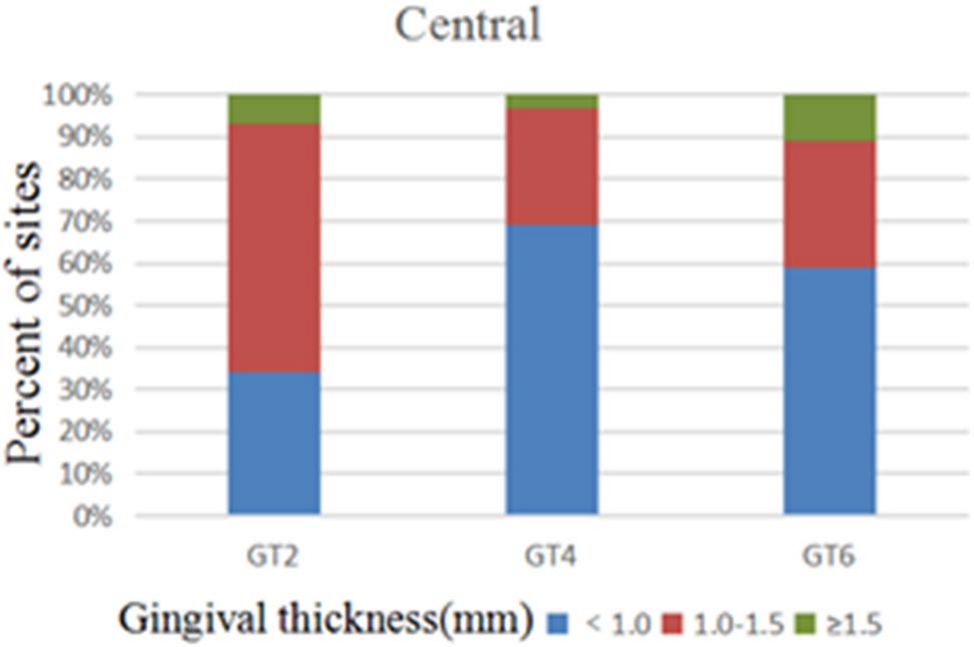
Distribution frequency of gingival thickness at different sites in the central incisor region.

**Figure 12:**
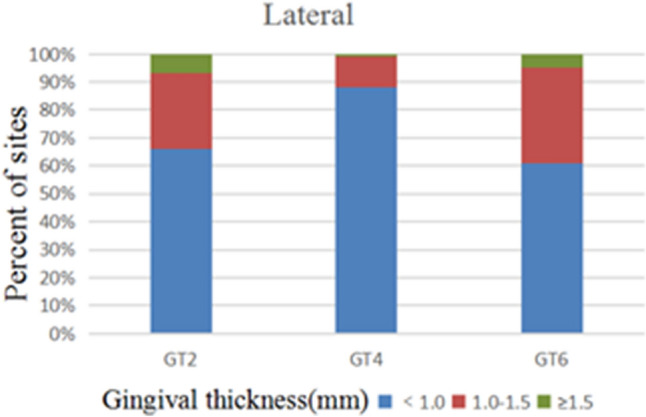
Distribution frequency of gingival thickness at different sites in the lateral incisor region.

**Figure 13:**
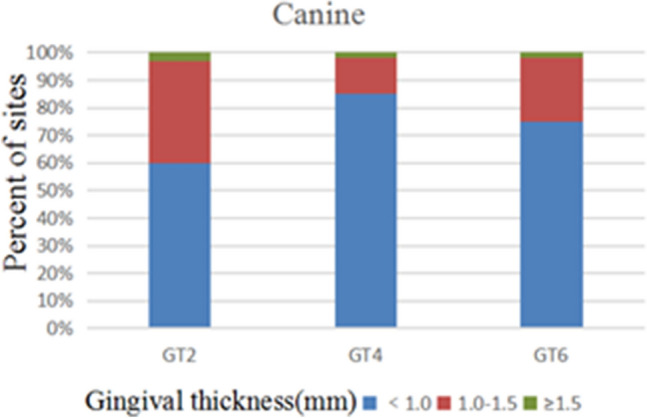
Distribution frequency of gingival thickness at different sites in the canine region.

1. **The correlation between BT and GT when measured by M1**
  The correlation between BT and GT measured by M1 is illustrated in Table 2. In 172 anterior teeth, the mean thickness of the underlying labial bone was less than 1.0 mm, whereas the mean GT was less than 1.5 mm. A strong negative correlation between BT and GT was identified at 2 mm apical to the CEJ in all maxillary anterior teeth, and a weak negative correlation at 4 mm apical to CEJ in CA. There was no correlation between BT and GT at 6 mm apical to the CEJ in the three types of teeth and 4 mm apical to the CEJ in CI and LI.
2. **The correlation between BT and GT when measured by M2**
  The correlation between BT and GT measured by M2 is presented in Table 3. There was a strong negative correlation between BT and GT at 2 mm apical to the CEJ in the three types of teeth. However, there was no correlation between BT and GT at other sites in the three types of teeth.
3. **Comparison of the buccal bone and gingival thickness to tooth type**
  There was a significant difference in the mean thickness of the buccal bone in the studied teeth by M1 (P < 0.001) and M2 (P < 0.001). The BT of the CI was greater than those of LI and CA. Concerning GT, a difference was also found among the three types of tooth, and the thinnest GT was observed in CA (Table 4).
4. **Distribution frequency of the labial BT and GT at different dental positions and sites in M2**
  Analysis of the distribution frequency in labial BT measurements at 2, 4, and 6 mm apical to the CEJ of the three types of teeth is depicted in Figs. 8, 9, 10, 11, 12, 13. For CI, 20 (11.63%) sites had a thickness less than 0.5 mm, 142 (82.56%) sites had a thickness of 0.5–1 mm, 6 (3.43%) sites had a thickness of 1.0–1.5 mm, and no sites had a thickness of more than 1.5 mm. For LI, 91 (52.91%) sites had a thickness of 0.5–1.0 mm, 4 (2.33%) sites had a thickness of 1.0–1.5 mm, 73 (42.44%) sites had a thickness less than 0.5 mm, and no sites exhibited a thickness of 1.5 mm or more. For CA, 163 (90.56%) sites were less than 1.0 mm thick, 14 (7.78%) sites were 1.0–1.5 mm thick, and only 3 (1.67%) sites were more than 1.5 mm thick.
  As for the GT, at the CI site, 90 (52.33%) sites had a thickness less than 1.0 mm, 66 (38.37%) sites had a thickness of 1.0–1.5 mm, and 12 (6.98%) sites had a thickness greater than 1.5 mm. For LI, 120 (69.77%) sites had a thickness less than 1.0 mm, and 40 (23.26%) sites were 1.0–1.5 mm thick. A total of 8 (4.65%) sites had a thickness greater than 1.5 mm. For CA, 132 (73.33%) sites were less than 1.0 mm, 44 (24.44%) sites were 1.0–1.5 mm, and 4 (2.22%) sites more than 1.5 mm were detected.
5. **Comparison between M1 and M2**
  The comparison between M1 and M2 on BT and GT at 2 mm below the CEJ was analyzed by the paired t-test (Table 5). There was no statistical difference in BT measured by M1 and M2 at 2 mm below CEJ of the three types of teeth. However, a significant difference in GT was found between M1 and M2, suggesting that caution is warranted when assessing GB during clinical procedures.

**Table 1:** Internal consistency between the two measurements.

Variables	ICC (95% CI)
BT	0.865 (0.809, 0.906)
GT	0.813 (0.738, 0.866)

**Table 2:** Gingival thickness (GT) and bone thickness (BT) at 2, 4, and 6 mm apical to the CEJ by M1.

Level	Variables	Central incisors(CI)	Lateral incisors(LI)	Canines (CA)
At 2 mm below the CEJ	GT (mm)	1.27 ± 0.31	1.02 ± 0.29	1.13 ± 0.34
	BT (mm)	0.54 ± 0.33	0.44 ± 0.29	0.42 ± 0.38
	*r*	− 0.823**	− 0.747**	− 0.655**
At 4 mm below the CEJ	GT (mm)	1.05 ± 0.20	0.89 ± 0.29	0.85 ± 0.35
	BT (mm)	0.93 ± 0.17	0.78 ± 0.27	0.92 ± 0.35
	*r*	− 0.017	− 0.261	− 0.377*
At 6 mm below the CEJ	GT (mm)	1.16 ± 0.37	1.14 ± 0.26	0.92 ± 0.30
	BT (mm)	0.77 ± 0.21	0.40 ± 0.35	0.73 ± 0.35
	*r*	0.201	0.101	− 0.061

*Confidence levels (bilateral) < 0.05 indicated a significant correlation.

**Confidence levels (bilateral) of < 0.01 indicated a significant correlation;

**Table 3:** GT and BT at 2, 4, and 6 mm apical to the CEJ measured by M2.

Level	Variables	Central incisors(CI)	Lateral incisors(LI)	Canines(CA)
At 2 mm below the CEJ	GT (mm)	1.10 ± 0.25	0.91 ± 0.25	0.94 ± 0.27
	BT (mm)	0.52 ± 0.22	0.40 ± 0.22	0.41 ± 0.37
	*r*	− 0.691**	0.676**	− 0.631**
At 4 mm below the CEJ	GT (mm)	0.86 ± 0.17	0.76 ± 0.23	0.73 ± 0.29
	BT (mm)	0.75 ± 0.11	0.66 ± 0.20	0.77 ± 0.29
	*r*	− 0.071	− 0.264	− 0.226
At 6 mm below the CEJ	GT (mm)	0.98 ± 0.32	0.97 ± 0.28	0.82 ± 0.20
	BT (mm)	0.66 ± 0.11	0.37 ± 0.22	0.59 ± 0.28
	*r*	0.214	0.281	− 0.280

**Confidence levels (bilateral) of < 0.01 indicated a significant correlation.

**Table 4:** The mean ± standard deviation of GT and BT (mm) in maxillary anterior teeth.

Tooth types	M1		M2	
	GT (mm)	BT (mm)	GT (mm)	BT (mm)
CI	1.16 ± 0.31	0.74 ± 0.29	0.98 ± 0.27	0.64 ± 0.18
LI	1.01 ± 0.29	0.54 ± 0.35	0.88 ± 0.27	0.48 ± 0.25
CA	0.96 ± 0.90	0.69 ± 0.41	0.83 ± 0.24	0.59 ± 0.34
*P*-value	< 0.001	< 0.001	< 0.001	< 0.001

**Table 5:** Comparison between M1 and M2 on BT and GT.

Level		Central incisors(CI)	Lateral incisors(LI)	Canines(CA)
BT	M1 (mm)	0.54 ± 0.33	0.44 ± 0.29	0.42 ± 0.38
	M2 (mm)	0.52 ± 0.22	0.40 ± 0.22	0.41 ± 0.37
	*t*	0.478	0.888	0.006
	*P*-value	0.636	0.382	0.995
GT	M1 (mm)	1.27 ± 031	1.02 ± 0.29	1.13 ± 0.34
	M2 (mm)	1.10 ± 0.25	0.91 ± 0.25	0.94 ± 0.27
	*t*	3.531	2.736	3.191
	*P*-value	0.001	0.011	0.003

## Discussion

Herein, the correlation between labial GT and BT in the maxillary anterior region was evaluated. A significant negative correlation was identified between BT and GT at 2 mm apical to the CEJ by M1 and M2 in all maxillary anterior teeth, whereas there was a weak negative correlation at 4 mm apical to the CEJ for CA by M1. No significant correlation was found at other sites both by M1 and M2. This finding is consistent with the results of other studies, such as Cao et al., who used a new method of CBCT combined with indirect gingival imaging to analyze the relationship between labial soft and hard tissues of the maxillary anterior teeth. Their study reported that GT at the site 2 mm below the labial central alveolar ridge in the upper anterior region was negatively correlated with BT^20^. Likewise, Stein et al. also discovered a negative correlation between BT and GT in the proximal labial side of the maxillary anterior teeth using the transparency method with a gingival marginal periodontal probe^11^. However, Fu et al. measured the thickness of soft and hard tissues among maxillary anterior teeth (2 mm apical to the alveolar crest) using a caliper and an imaging method. In contrast, they found a positive correlation between soft tissue thickness and the underlying bone^6^. This discrepancy among researchers highlights the controversial correlation between the thicknesses of soft and hard tissues that commonly exist and are applied in preoperative planning. Some reports speculate that this inconsistency among research may be related to different measuring methods, diverse phenotypes in patients' ethnicity, and differences in the measuring position^6,11,20^. However, the results of this study further denote that the clinical evaluation of esthetic areas is complex for implantation timing (either immediate, early, or delayed) and for indications of esthetic risk (such as gingival recession or detachment). Furthermore, individual methods of judging soft tissue biotypes for esthetic risk using only the naked eye are unreliable.

Multiple studies have established that GB is a comprehensive result of several factors, not only the thickness of the gingiva but also the thickness of the underlying bone, the dimensions of the dentogingival complex, the morphology of the crown, the bone crest of the cemento-enamel junction, and so on^9,10,33,34^. GT in conjunction with the underlying BT showed the highest correlation with GB. The standard method for determining GT is transparency probing by a periodontal probe. However, other factors must also be considered when assessing GB, such as the shape of the underlying bone and the size of the teeth. Furthermore, GT can decrease due to aging and other factors and is the leading cause of periodontal detachment and gingival retraction^35^. Previous studies have corroborated that the risk of gingival retraction in patients with thin GB was significantly higher than patients with thick GB in periodontal and implantation treatments^36^, and GB can be adjusted by surgical techniques such as gingival grafting or subepithelial connective tissue grafting to augment soft tissues^37,38^.

Due to the aforementioned inconsistencies in the determination of GB, clinical evaluations for indications of immediate implantation cannot solely rely on the transparency probing technique. Eghbali et al. discussed the unreliability of visual examination for measuring GB. The study postulated that roughly half of the participating patients with thin gingiva had been mistakenly classified as other biotypes^39^. Kan et al. used calipers to directly measure GT at the point 2 mm under the middle margin of the extraction sockets, and the result determined that gingivae < 1 mm should be designated as thin biotypes, while those > 1 mm should be defined as thick biotypes^10^. Claffey et al. measured GT at the point 2 mm submarginal by puncture and subsequently proposed that 1.5 mm would suffice as the maximal value for thin biotypes while gingivae > 2 mm should be designated thick GB^40^.

In this study, BT measured by M1 was consistent with M2 at 2 mm below the CEJ of the three types of teeth. However, significant differences was found between GT measured by CBCT imaging alone and CBCT imaging matched with DIS, the value measured by digital scanning combined with CBCT was significantly smaller than CBCT alone at 2 mm below the CEJ. Although the superposition of oral scan imaging and CBCT increased the visibility of the gingival contour, there was no evidence of the accuracy of this method in the measurement of soft tissue thickness, strongly suggesting that caution is required when assessing GB during clinical procedures.

As for the frequency distribution of buccal bone and gingival thickness, M2 results exhibited that the proportion of GT > 1.5 mm was 7.1% for CI, 4.8% for LI, and 2.2% for CA. The proportion of BT for maxillary anterior teeth > 1 mm was also significantly lower, with proportions of the thick biotype for the CI, LI, and CA of 3.3%, 2%, and 9%, respectively. The percentage of people who meet the criteria for immediate implantation in the esthetic area is very low, according to Buser et al.^1^. Clinical recommendations regarding the immediate implantation of anterior teeth should be made with great caution. Most notably, this study found significant negative correlations between GT and BT at 2 mm apical to the CEJ in the anterior tooth region. Therefore, when evaluating the esthetic risks prior to implantation in anterior regions, it will be ill-advised to justify that a thick GB necessarily corresponds to a thick gingiva and bone plate since this often leads to incorrect clinical decisions.

## Conclusions

Within the limitations of this study, we demonstrated a negative correlation between labial GT and BT in the anterior region at the site 2 mm apical to the CEJ. A thin labial BT and GT (< 1 mm) may be expected in over half of the maxillary anterior teeth. It should be noted that thick gingiva does not necessarily correspond to a thick underlying bone plate in clinical practice.
